# Impaired episodic-like memory in a mouse model of Alzheimer's disease is associated with hyperactivity in prefrontal–hippocampal regions

**DOI:** 10.1242/dmm.049945

**Published:** 2023-03-10

**Authors:** Sijie Tan, Wen Han Tong, Ajai Vyas

**Affiliations:** ^1^ School of Biological Sciences, Nanyang Technological University, Singapore 637551; ^2^ Lee Kong Chian School of Medicine, Nanyang Technological University, Singapore 308232

**Keywords:** APP knock-in, Dementia, Episodic memory, Hippocampus, Medial prefrontal cortex, Preclinical AD

## Abstract

Alzheimer's disease (AD) is a degenerative brain disorder with a long prodromal period. An APP^NL-G-F^ knock-in mouse model is a preclinical model to study incipient pathologies during the early stages of AD. Despite behavioral tests revealing broad cognitive deficits in APP^NL-G-F^ mice, detecting these impairments at the early disease phase has been challenging. In a cognitively demanding task that assessed episodic-like memory, 3-month-old wild-type mice could incidentally form and retrieve ‘what–where–when’ episodic associations of their past encounters. However, 3-month-old APP^NL-G-F^ mice, corresponding to an early disease stage without prominent amyloid plaque pathology, displayed impairment in recalling ‘what–where’ information of past episodes. Episodic-like memory is also sensitive to the effect of age. Eight-month-old wild-type mice failed to retrieve conjunctive ‘what–where–when’ memories. This deficit was also observed in 8-month-old APP^NL-G-F^ mice. c-Fos expression revealed that impaired memory retrieval in APP^NL-G-F^ mice was accompanied by abnormal neuronal hyperactivity in the medial prefrontal cortex and CA1 dorsal hippocampus. These observations can be used for risk stratification during preclinical AD to detect and delay the progression into dementia.

## Teaser

**Summary:** Using a preclinical Alzheimer's disease model, our work highlights that episodic memory impairment at early disease stage is associated with neuronal hyperactivity in specific medial prefrontal cortex and hippocampus regions.

## INTRODUCTION

Alzheimer's disease (AD) is the most common cause of dementia. The neuropathology of AD follows a cascade of events initiating from amyloid-beta (Aβ) and hyperphosphorylated tau depositions, inflammatory reactions, and subsequent synaptic and neuronal loss, which begin to appear before clinical diagnosis (Duyckaerts et al., 2009). Patients typically display no significant symptoms in this preclinical phase apart from occasional memory lapses (Rabin et al., 2017). Subsequently, the neuropathology culminates in neurodegeneration and the onset of severe memory and cognitive impairments.

Animal models of AD are excellent tools to study disease development, including offering insights into the early disease stage. Traditionally, AD research relies on transgenic mouse models overexpressing amyloid precursor protein (APP) to recapitulate features of human AD (Sasaguri et al., 2017). However, the random APP transgene insertion and overproduction of APP can lead to extraneous phenotypes unrelated to AD (Sasaguri et al., 2017). An alternative APP mouse model was generated to evade this problem (Saito et al., 2014). A knock-in strategy was used to ‘humanize’ the murine Aβ sequence and to introduce three familial AD mutations [Swedish (NL), Arctic (G), Beyreuther/Iberian (F)] into the murine APP locus (APP^NL-G-F^) (Saito et al., 2014). APP^NL-G-F^ mice exhibit age-dependent deposition of Aβ plaques and neuroinflammation in the cortical and subcortical regions of the brain (Saito et al., 2014; Masuda et al., 2016; Whyte et al., 2018; Latif-Hernandez et al., 2019; Mehla et al., 2019b) but do not suffer from neurodegeneration (Sasaguri et al., 2017). The absence of severe neuronal loss makes the APP^NL-G-F^ an excellent preclinical AD mouse model.

APP^NL-G-F^ mice show Aβ plaque deposition in the brain as early as 4-6 months of age (Masuda et al., 2016; Latif-Hernandez et al., 2019; Mehla et al., 2019b; Saifullah et al., 2020). However, classical behavioral tasks could only detect cognitive deficits at later ages (Masuda et al., 2016; Whyte et al., 2018; Sakakibara et al., 2018; Latif-Hernandez et al., 2019; Mehla et al., 2019b; Pervolaraki et al., 2019). The lack of discernable memory and cognitive changes in young APP^NL-G-F^ mice does not reflect the lack of deficits in the early disease phase. Instead, experimental paradigms may not be sensitive enough to detect such alterations in young animals (Saifullah et al., 2020). A behavioral task that places greater demands on cognitive capacity and flexibility could potentially capture memory impairments in young APP^NL-G-F^ mice. Detection of memory and cognitive alterations at an early stage during AD progression is critical because it allows for timely stratification and therapeutic interventions before deficits are widespread and irreversible.

In this study, we sought to detect memory deficits in APP^NL-G-F^ mice during the early disease stage by using a behavioral task that assesses a higher-order memory function – episodic memory. Episodic memory is a long-term memory that requires multi-modal integration of contextual (‘what’), spatial (‘where’) and temporal (‘when’) information (Tulving, 1983). Tapping on the mice's spontaneous exploratory behavior towards environmental cues, and innate scent-marking behavior known to be activated only by the presence of another mouse (Arakawa et al., 2008b), we proposed a behavioral paradigm that quantitatively assessed whether mice could retrieve distinct episodic associations based on their brief encounters with an object or a female mouse (‘what’) at a defined location (‘where’) and at a specific time of the day (‘when’). We studied episodic-like memory retrieval in 3- and 8-month-old APP^NL-G-F^ mice, corresponding to the early and late disease stage before and after significant Aβ plaque deposition, respectively (Masuda et al., 2016; Latif-Hernandez et al., 2019; Mehla et al., 2019b; Saifullah et al., 2020). Analogous to human episodic memory, episodic-like memory representation in rodents is highly dependent on the medial prefrontal cortex (mPFC) and hippocampus (Chao et al., 2020). With this, we imaged the expression of the immediate early gene product c-Fos (also known as Fos), a marker for neuronal activity expressed during learning and memory (Tischmeyer and Grimm, 1999), in the mice after episodic information retrieval to study whether memory performance was linked to neural activity changes in the mPFC and hippocampus regions. Our study is the first to characterize an age-dependent episodic-like memory decline in APP^NL-G-F^ mice and to detect the deficit early before prominent Aβ plaque deposition. In addition, our work suggests that perturbed neural activity in specific prefrontal–hippocampal regions could underlie poor episodic-like memory retrieval in APP^NL-G-F^ mice.

## RESULTS

### Age-dependent Aβ deposition in the brain of APP^NL-G-F^ mice

APP^NL-G-F^ mice exhibit age-dependent Aβ deposition in the cortex and hippocampus (Saito et al., 2014; Masuda et al., 2016; Whyte et al., 2018; Latif-Hernandez et al., 2019; Mehla et al., 2019b). To confirm the pattern of Aβ deposition during disease progression, brain sections from 3- and 8-month-old wild-type (WT) and APP^NL-G-F^ mice were stained with anti-Aβ_1-42_, which recognizes the soluble and insoluble forms of Aβ peptides. Aβ was hardly detectable in WT mice but became increasingly apparent across the brain hemisphere with age in the APP^NL-G-F^ mice (Fig. S2A). In 3-month-old APP^NL-G-F^ mice, significantly higher Aβ levels were observed in the cortex and CA1 dorsal hippocampus compared to those in the age-matched WT mice (Fig. S2B,C). In 8-month-old APP^NL-G-F^ mice, significantly higher Aβ levels were detected in the cortex and hippocampus compared to those in the age-matched WT mice (Fig. S2B-E). In addition, a comparison between the 3- and 8-month-old APP^NL-G-F^ revealed significantly higher Aβ across the brain regions in 8-month-old animals (Fig. S2B-E). These observations highlight a progressive accumulation of Aβ with age in APP^NL-G-F^ mice.

### Three-month-old WT mice can retrieve ‘what–where–when’ episodic associations of past experiences

**Fig. 1.**
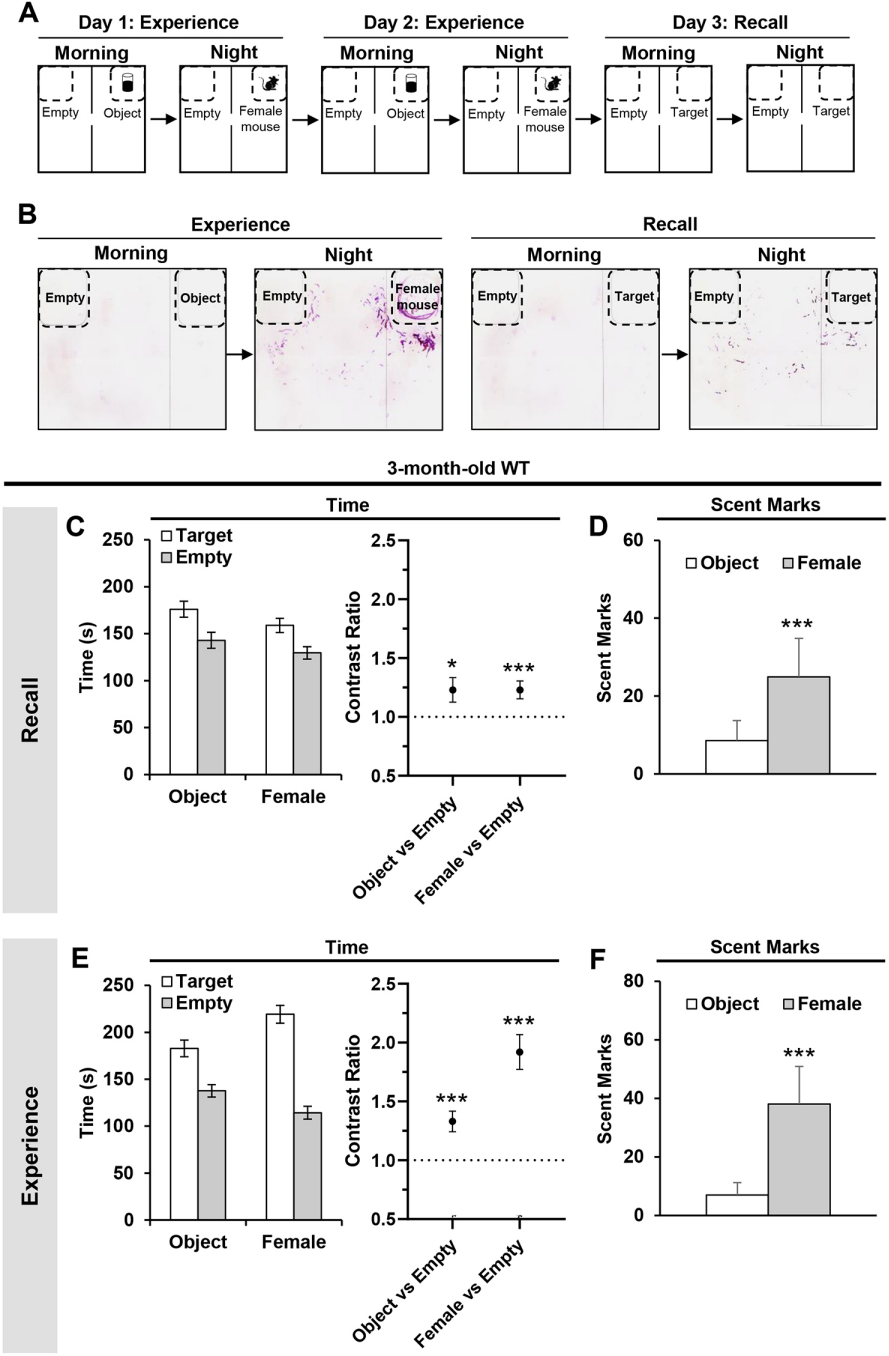
**Intact retrieval of ‘what–where–when’ episodic-like memories in 3-month-old WT mice.** (A) Sequence of the ‘what–where–when’ episodic-like memory behavioral task. (B) Expected scent-marking patterns (purple streaks) produced by the mouse if episodic-like memory retrieval was intact. The representative scent-marking patterns were from one of the 3-month-old WT mice that had undergone the behavioral task. (C) Absolute time spent around each zone (left) and the corresponding contrast ratios (right) during the recall tests. Contrast ratios were calculated as the proportion of time spent around the target relative to the empty zone. (D) Absolute scent marks during the recall tests at the time of object and female mouse encounters. (E) Absolute time spent (left) and the corresponding contrast ratios (right) during the second experience trials. (F) Absolute scent marks around the object and female mouse during the second experience trials. For C and E contrast ratio graphs, statistical analysis was performed with a nested generalized linear mixed model (GLMM) with a gamma distribution. Time:(Zone)+(1|Animal)*.* ‘Zone’ refers to either the target (object or female mouse) or the corresponding empty zone. For D and F, statistical analysis was performed with Wilcoxon signed rank test. All values are mean±s.e.m. *P≤0.05 and ***P≤0.005. 3-month-old WT mice (*n*=27). Each animal was used as a biological replicate.

The principle of the task was to briefly confront the mice with two distinct episodes in a day. In each episode, an object or a female mouse (‘what’) was present in a corner of an arena (‘where’) depending on the time of the day (‘when’) (Fig. 1A). The mice encountered these episodes for two consecutive days to encourage them to learn the episodic associations (‘experience’ phase) (Fig. 1A). On the third day, the mice were tested on their ability to retrieve this episodic information in an empty (no object or female mouse presence) but otherwise familiar arena (‘recall’ phase) (Fig. 1A). The mice's exploratory and scent-marking responses were used to quantitatively evaluate episodic-like memory retrieval. Exploratory behavior indicates whether the mice remembered the target location inside the arena (‘what–where’ memory; spatial memory). An intact recollection would be reflected as the mice spending more time exploring the target compared to the opposing empty zone. Scent-marking behavior, which would be activated by the memory of a female mouse but not the object, indicates whether the mice could discriminate between the object and female mouse episodes or when the target appeared (‘what–when’ memory; temporal memory). An intact recall would be reflected as the mice scent marking more around the target during the diurnal phase tied to the female mouse compared to the object (Fig. 1B). Expression of these appropriate exploratory and scent-marking responses during the recall tests represents successful binding of spatial and temporal information or ‘what–where–when’ information, also known as episodic-like memory.

In the recall tests, 3-month-old WT mice spent more time around the target zone compared to the opposing empty zone at the time of object and female mouse encounters [*t*_(50)_=2.431, *P*=0.0187 for object versus empty; *t*_(50)_=3.296, *P*=0.0018 for female versus empty; generalized linear mixed model (GLMM) with gamma distribution] (Fig. 1C; left graph represents the absolute time spent and right graph indicates the contrast ratios). Three-month-old WT mice also scent marked more around the target zone during the time of the day associated with the female mouse compared to that of the object (*P*<0.0029; Wilcoxon test) (Fig. 1D). To confirm that the spatial preferences and scent-marking responses during the recall tests were based on memory retrieval, we evaluated the exploratory and scent-marking responses of the mice during the preceding experience phase. In the experience trials, the mice spent more time near the object and the female mouse compared to the opposing empty zone [*t*_(50)_=4.338, *P*=0.0001 for object versus empty; *t*_(50)_=8.492, *P*<0.0001 for female versus empty; GLMM with gamma distribution] (Fig. 1E). The mice also scent marked more around the female mouse compared to the object (*P*=0.001, Wilcoxon test) (Fig. 1F). These behavioral responses in the experience phase, combined with the similar responses observed in the recall tests, highlight that 3-month-old WT mice can retrieve conjunctive ‘what–where–when’ information about their past experiences.

### Three-month-old APP^NL-G-F^ mice exhibit a deficit in recollecting ‘what–where’ information of past episodes

**Fig. 2.**
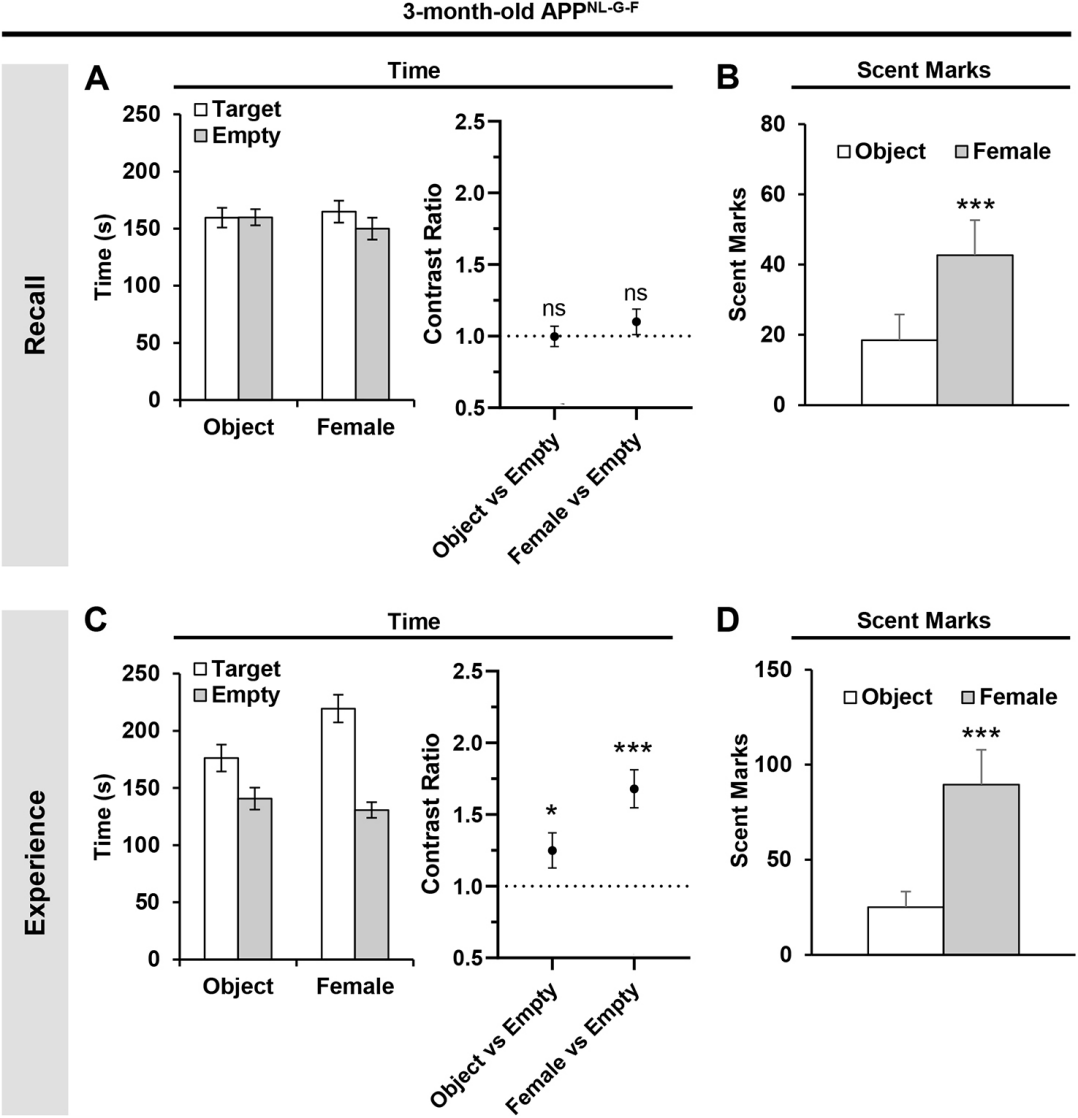
**Impaired retrieval of ‘what–where’ information of the past episodes in 3-month-old APP^NL-G-F^ mice.** (A) Absolute time spent (left) and the corresponding contrast ratios (right) during the recall tests. (B) Absolute scent marks during the recall tests at the time of object and female mouse encounters. (C) Absolute time spent (left) and the corresponding contrast ratios (right) during the second experience trials. (D) Absolute scent marks around the object and female mouse during the second experience trials. Statistical analyses were performed with a nested GLMM with a gamma distribution (A,C contrast ratio graphs) or Wilcoxon signed rank test (B,D). All values are mean±s.e.m. *P≤0.05 and ***P≤0.005; ns, not significant. 3-month-old APP^NL-G-F^ mice (*n*=29). Each animal was used as a biological replicate.

Given that 3-month-old WT mice can retrieve unique episodic-like memories (Fig. 1), we examined whether the cognitive ability was perturbed in age-matched APP^NL-G-F^ mice. In the recall tests, 3-month-old APP^NL-G-F^ mice did not display a preference to explore the target zone compared to the opposing empty zone at the time of object and female mouse encounters [*t*_(54)_=−0.027, *P*=0.9789 for object versus empty; *t*_(54)_=1.16, *P*=0.2511 for female versus empty; GLMM with gamma distribution] (Fig. 2A). The lack of spatial preferences was not due to compromised exploratory behavior in the 3-month-old APP^NL-G-F^ mice; these mice spent more time around the object and female mouse compared to the opposing empty zone in the preceding experience trials [*t*_(54)_=2.276, *P*=0.0271 for object versus empty; *t*_(54)_=6.557, *P*<0.0001 for female versus empty; GLMM with gamma distribution] (Fig. 2C). Hence, the lack of spatial preferences by the 3-month-old APP^NL-G-F^ mice during the recall tests was due to impaired retrieval of ‘what–where’ information of past episodes. However, analysis of the scent marks in the recall tests showed that the mice scent marked significantly more around the target zone during the time of the day associated with the female mouse compared to that of the object (*P*=0.0031, Wilcoxon test) (Fig. 2B). A similar response was observed in the preceding experience trials (*P*<0.0001; Wilcoxon test) (Fig. 2D), highlighting that recollection of ‘what–when’ information was intact in the 3-month-old APP^NL-G-F^ mice.

### Eight-month-old WT mice exhibit a deficit in retrieving ‘what–where–when’ episodic-like memories

Because a partial deficit in episodic-like memory retrieval was detected in 3-month-old APP^NL-G-F^ mice (Fig. 2), we examined whether the phenotype was further aggravated in 8-month-old animals, in which the Aβ pathology was more aggressive (Fig. S2). First, we investigated whether age itself affects episodic-like memory by studying the 8-month-old WT mice.

**Fig. 3.**
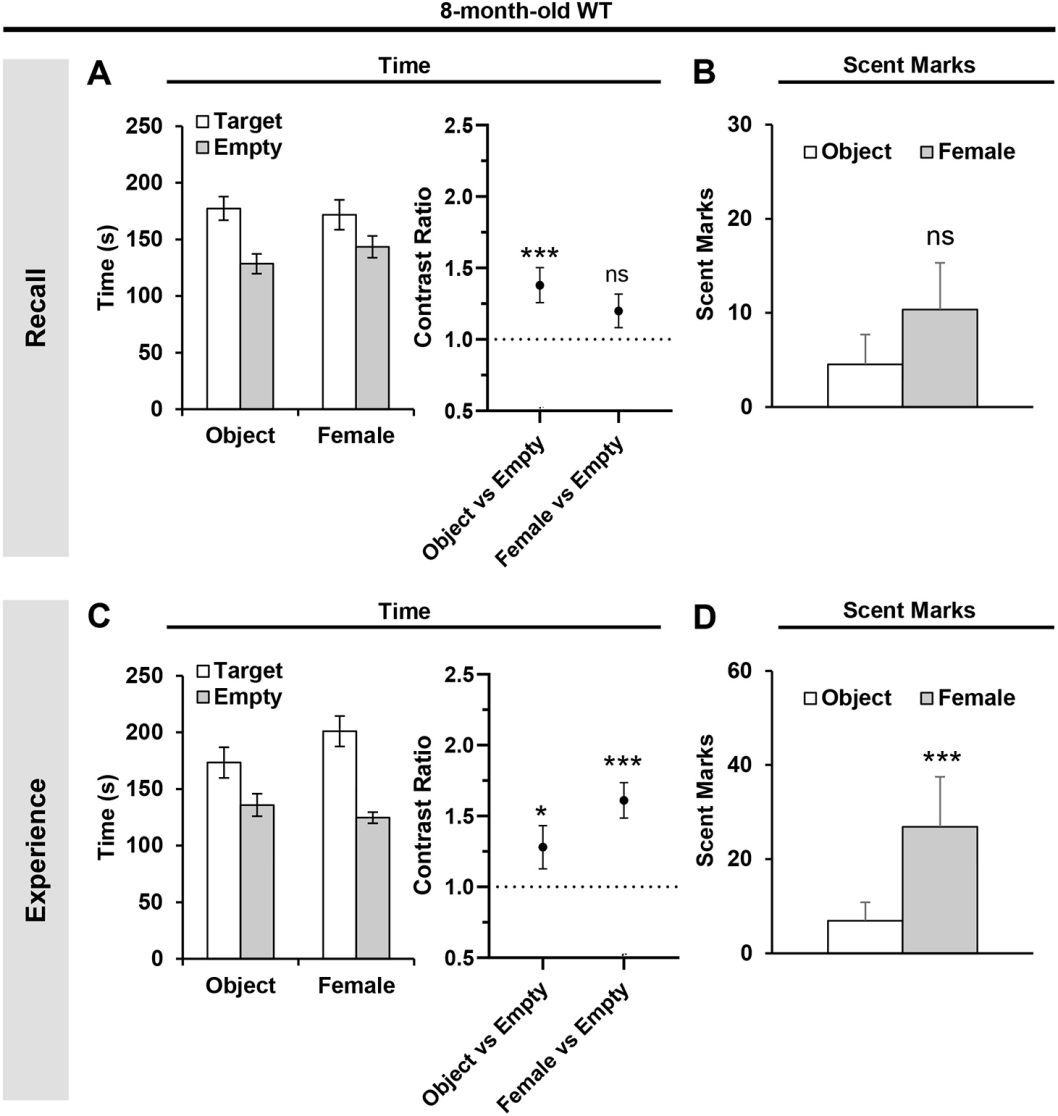
**Impaired retrieval of ‘what–where–when’ episodic-like memories in 8-month-old WT mice.** (A) Absolute time spent (left) and the corresponding contrast ratios (right) during the recall tests. (B) Absolute scent marks during the recall tests at the time of object and female mouse encounters. (C) Absolute time spent (left) and the corresponding contrast ratios (right) during the second experience trials. (D) Absolute scent marks around the object and female mouse during the second experience trials. Statistical analyses were performed with a nested GLMM with a gamma distribution (A,C contrast ratio graphs) or Wilcoxon signed rank test (B,D). All values are mean±s.e.m. *P≤0.05 and ***P≤0.005; ns, not significant. 8-month-old WT mice (*n*=19). Each animal was used as a biological replicate.

In the recall tests, 8-month-old WT mice spent more time around the object-target zone but did not show a preference for the female mouse-target zone [*t*_(34)_=3.605, *P*=0.001 for object versus empty; *t*_(34)_=1.83, *P*=0.076 for female versus empty; GLMM with gamma distribution] (Fig. 3A). In addition, the mice did not show preferential scent-marking behavior during the time of the day linked to the female mouse versus that of the object (*P*=0.2969, Wilcoxon test) (Fig. 3B). These observations highlight a probable deficit in retrieving ‘what–where–when’ episodic associations in 8-month-old WT mice. To confirm the impairment, we evaluated the exploratory and scent-marking behaviors of these mice in the preceding experience phase. Eight-month-old WT mice preferentially spent more time exploring the object and the female mouse compared to the opposing empty zone in the experience trials [*t*_(34)_=2.04, *P*=0.0492 for object versus empty; *t*_(34)_=6.175, *P*<0.0001 for female versus empty; GLMM with gamma distribution] (Fig. 3C). These mice also scent marked more around the female mouse compared to the object (*P*=0.00039; Wilcoxon test) (Fig. 3D). Hence, the lack of spatial preference (at least for the female mouse target zone) (Fig. 3A) and target recognition (Fig. 3B) by the 8-month-old WT mice during the recall tests was not due to impaired exploratory and scent-marking behaviors, but a deficit in retrieving of ‘what–where–when’ episodic associations of past experiences.

### Eight-month-old APP^NL-G-F^ mice exhibit a deficit in recollecting ‘what–where–when’ episodic-like memories

**Fig. 4.**
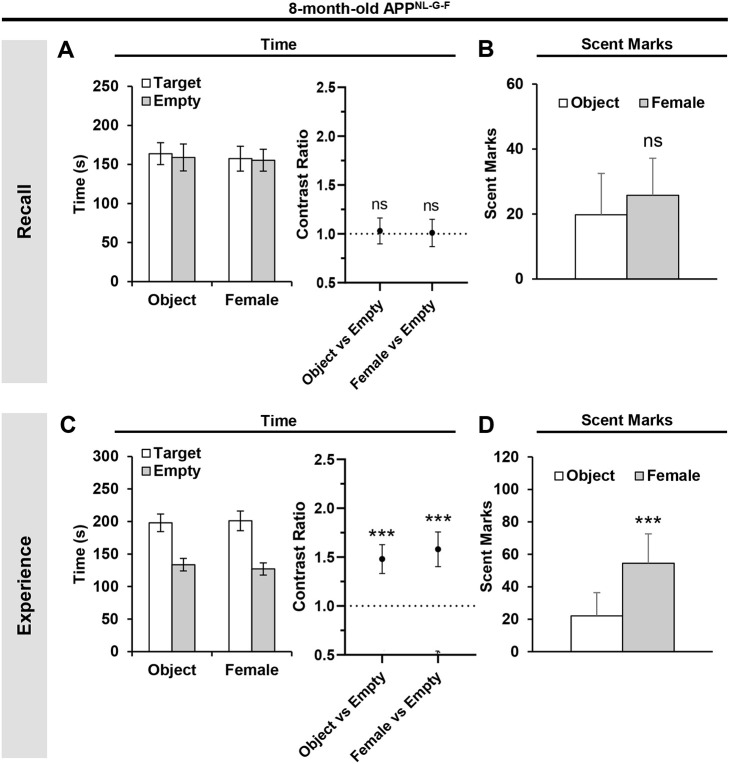
**Impaired retrieval of ‘what–where–when’ episodic-like memories in 8-month-old APP^NL-G-F^ mice.** (A) Absolute time spent (left) and the corresponding contrast ratios (right) during the recall tests. (B) Absolute scent marks during the recall tests at the time of object and female mouse encounters. (C) Absolute time spent (left) and the corresponding contrast ratios (right) during the second experience trials. (D) Absolute scent marks around the object and female mouse during the second experience trials. Statistical analyses were performed with a nested GLMM with a gamma distribution (A,C contrast ratio graphs) or Wilcoxon signed rank test (B,D). All values are mean±s.e.m. ***P≤0.005; ns, not significant. 8-month-old APP^NL-G-F^ mice (*n*=13). Each animal was used as a biological replicate.

Given that age had a profound influence on episodic-like memory retrieval in WT mice (Fig. 3), we examined whether the deficit was aggravated in 8-month-old APP^NL-G-F^ mice. In the recall tests, these mice did not display a preference to explore the target zone compared to the opposing empty zone at the time of object and female mouse encounters [*t*_(22)_=0.24, *P*=0.8129 for object versus empty; *t*_(22)_=0.09, *P*=0.929 for female versus empty; GLMM with gamma distribution] (Fig. 4A). In addition, these mice did not display asymmetrical scent-marking responses for the time of the day tied to the female mouse versus the object (*P*=0.1719; Wilcoxon test) (Fig. 4B). In the preceding experience phase, 8-month-old APP^NL-G-F^ mice spent more time around the female mouse and the object compared to the opposing empty zone [*t*_(22)_=3.963, *P*=0.0007 for object versus empty; *t*_(22)_=4.084, *P*=0.0005 for female versus empty; GLMM with gamma distribution] (Fig. 4C), and scent marked more around the female mouse compared to the object (*P*=0.0039; Wilcoxon test) (Fig. 4D). Hence, the lack of spatial preferences (Fig. 4A) and target recognition (Fig. 4B) by 8-month-old APP^NL-G-F^ mice during the recall tests was not due to impaired exploratory and scent-marking behaviors, but a deficit in retrieving ‘what–where–when’ episodic-like memories.

### Poor episodic-like memory retrieval in APP^NL-G-F^ mice is associated with hyperactivity in the mPFC and CA1 dorsal hippocampus

**Fig. 5.**
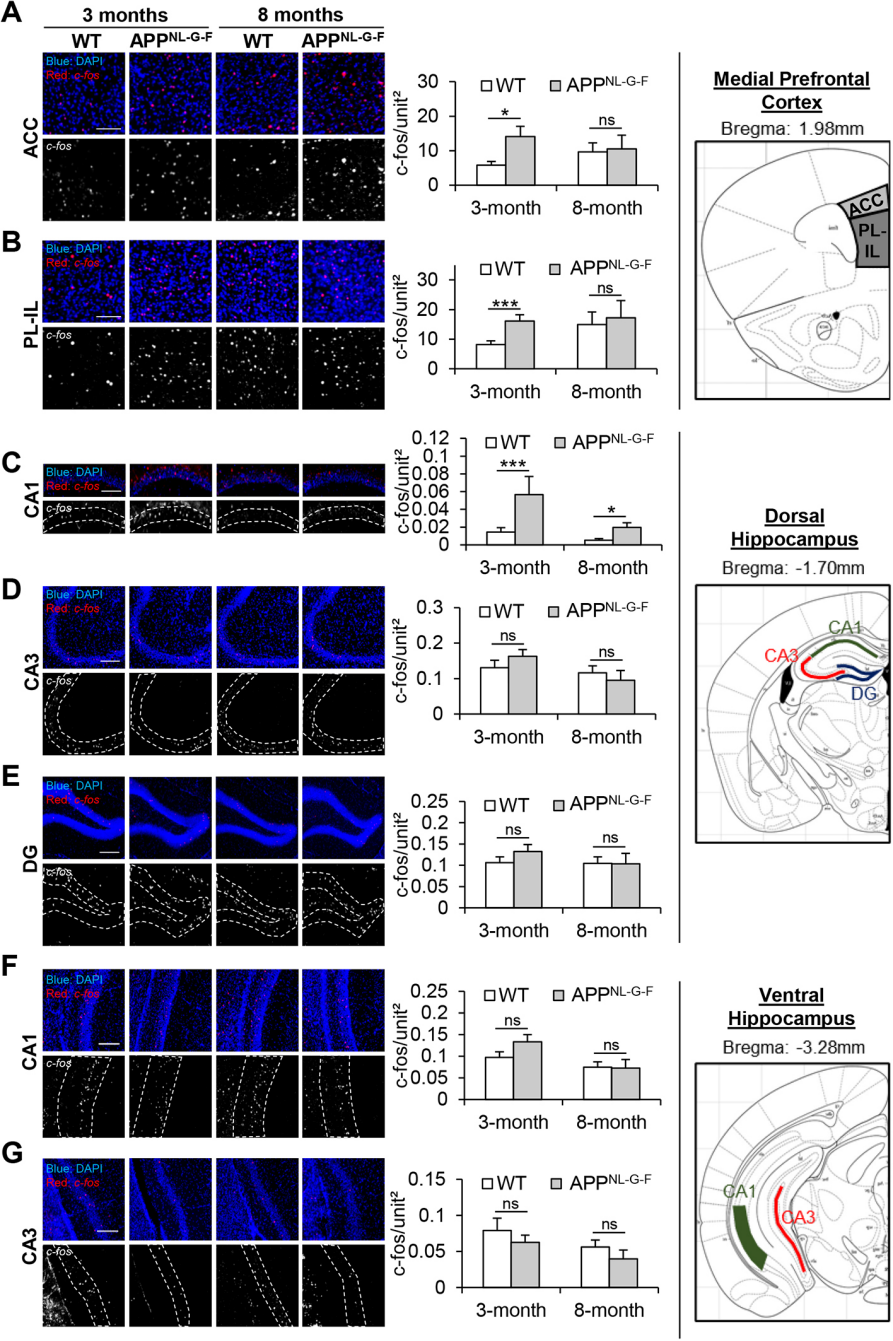
**c-Fos expression in the medial prefrontal cortex (mPFC) and hippocampus of WT and APP^NL-G-F^ mice after the memory recall tests.** (A-G) Number of c-Fos-positive puncta per unit area in the anterior cingulate cortex (ACC; A) and prelimbic–infralimbic (PL-IL) regions (B) of the mPFC; CA1 (C), CA3 (D) and dentate gyrus (DG; E) of the dorsal hippocampus; and CA1 (F) and CA3 (G) of the ventral hippocampus. Left: immunofluorescence images of merged c-Fos (red) and DAPI (blue) staining and extracted c-Fos-positive puncta in grayscale. White dashed lines in the grayscale images outline the brain regions. Scale bars: 100 µm. Middle: quantification of the number of c-Fos-positive puncta per unit brain area. Right: mouse brain coronal sections showing the respective brain regions. Images were from Paxinos and Franklin (2001). The mPFC image corresponds to the page at bregma 1.98 mm (figure 14), the dorsal hippocampus at bregma –1.70 mm (figure 42) and the ventral hippocampus at bregma −3.28 mm (figure 58). All values are mean±s.e.m. Statistical analysis was performed with Mann–Whitney *U-*test. *P≤0.05 and ***P≤0.005; ns, not significant. 3-month-old WT (*n*=22), 3-month-old APP^NL-G-*F*^ (*n*=24 for ACC, *n*=25 for remaining brain regions), 8-month-old WT (*n*=19), 8-month-old APP^NL-G-F^ (*n*=12). Each animal was used as a biological replicate.

The mPFC and hippocampus are critical for integrating ‘what–where–when’ information to construct episodic-like memory in rodents (Chao et al., 2020). Our study showed similar activation of the mPFC and hippocampus in mice following episodic-like memory retrieval with our behavioral paradigm (Fig. S3). The expression of c-Fos, a marker for neuronal activity, was significantly higher in the anterior cingulate cortex (ACC) (Fig. S3A), dorsal hippocampus (Fig. S3B) and ventral hippocampus (Fig. S3C) in mice that went through the episodic-like memory task (‘episodic’) compared to control mice that went through the behavior routine without the memory task (‘control’). Given the poor episodic-like memory retrieval in APP^NL-G-F^ mice (Figs 2 and 4), we examined whether neuronal activity in the mPFC and hippocampus of APP^NL-G-F^ mice was altered compared to that in the age-matched WT mice. In the ACC and prelimbic–infralimbic (PL-IL) regions of the mPFC, significantly higher c-Fos expression was observed in the 3-month-old APP^NL-G-F^ mice compared to the age-matched WT mice (*P*_ACC_=0.0235, *P*_PL-IL_=0.0029; Mann–Whitney test) (Fig. 5A,B). In contrast, no difference in c-Fos expression was observed between the 8-month-old WT and APP^NL-G-F^ mice (*P*_ACC_=0.9602, *P*_PL-IL_=0.8184; Mann–Whitney test) (Fig. 5A,B). In the CA1 dorsal hippocampus, higher c-Fos expression was observed in 3- and 8-month-old APP^NL-G-F^ mice compared to the age-matched WT mice (*P*_3month_=0.0036, *P*_8month_=0.0166; Mann–Whitney test) (Fig. 5C). For the CA3 (*P*_3month_=0.2750, *P*_8month_=0.4902; Mann–Whitney test) and dentate gyrus (DG; *P*_3month_=0.1640, *P*_8month_=0.7652; Mann–Whitney test) of the dorsal hippocampus, and the CA1 (*P*_3month_=0.2658, *P*_8month_=0.5353; Mann–Whitney test) and CA3 (*P*_3month_=0.4183, *P*_8month_=0.2196; Mann–Whitney test) of the ventral hippocampus, no differences in c-Fos expression were observed between the 3- and 8-month-old age-matched WT and APP^NL-G-F^ mice (Fig. 5D-G).

## DISCUSSION

Despite the early Aβ deposition in APP^NL-G-F^ mice, memory and cognitive deficits were only reported at later ages (Masuda et al., 2016; Whyte et al., 2018; Sakakibara et al., 2018; Latif-Hernandez et al., 2019; Mehla et al., 2019b; Pervolaraki et al., 2019). Here, we present a behavioral paradigm that could sensitively detect episodic-like memory impairment in APP^NL-G-F^ mice during the early disease stage. APP^NL-G-F^ mice at 3 and 8 months, corresponding to the disease stage before and after significant plaque pathology (Masuda et al., 2016; Latif-Hernandez et al., 2019; Mehla et al., 2019b; Saifullah et al., 2020), and their age-matched WT mice were confronted with two distinct episodes consisting of an object or a female mouse (‘what’) at a defined location (‘when’) and time (‘where’). These mice were assessed for their ability to remember the episodic associations. By analyzing the exploratory and scent-marking behaviors during the memory recall tests, our study shows that 3-month-old WT mice can modulate their behaviors based on intact retrieval of ‘what–where–when’ information of past episodes. In contrast, 3-month-old APP^NL-G-F^ mice displayed a deficit in recollecting ‘what–where’ target locations. Our study also highlights that episodic memory is sensitive to the effect of age, as 8-month-old WT mice displayed a profound impairment in retrieving conjunctive ‘what–where–when’ episodic-like memories. This deficit was also observed in 8-month-old APP^NL-G-F^ mice. c-Fos imaging shows that poor episodic-like memory retrieval in APP^NL-G-F^ mice is associated with neuronal hyperactivity in specific prefrontal–dorsal hippocampal regions.

In humans, preclinical AD lasts at least 20 years, when pathophysiological changes are initiated inside the brain before the onset of neurodegeneration and dementia (Bateman et al., 2012). The APP^NL-G-F^ mouse model is a preclinical model of AD. These mice exhibit age-dependent Aβ deposition in the cortex and hippocampus as early as 2 months old but do not suffer from neurodegeneration (Saito et al., 2014; Masuda et al., 2016; Whyte et al., 2018; Latif-Hernandez et al., 2019; Mehla et al., 2019b; Saifullah et al., 2020). Our study reports a similar Aβ pathology in APP^NL-G-F^ mice, where Aβ was found to accumulate progressively in the cortical and hippocampal regions from 3- to 8-month-old mice. The lack of neurodegeneration in the APP^NL-G-F^ mouse model is likely to be caused by the absence of tau pathology. Hence, the APP^NL-G-F^ is considered a preclinical AD model by recapitulating the long Aβ amyloidosis period before the induction of tauopathy and neurodegeneration associated with later AD stages (Sasaguri et al., 2017).

Most behavioral studies that evaluated memory and cognitive impairments in the APP^NL-G-F^ mice captured the deficits only at later ages despite the profound Aβ pathology in young animals (Masuda et al., 2016; Whyte et al., 2018; Sakakibara et al., 2018; Latif-Hernandez et al., 2019; Mehla et al., 2019b; Pervolaraki et al., 2019). There is evidence to suggest that the cognitive load of a behavioral task can influence its sensitivity to detect a memory alteration (Gidyk et al., 2015). For example, touchscreen-based location discrimination and paired associate learning tasks could sensitively detect spatial memory deficit in 4- to 6-month-old APP^NL-G-F^ mice; however, classical Morris water maze (MWM) did not reveal a difference between the control and APP^NL-G-F^ mice (Saifullah et al., 2020). In another study, a modified MWM with higher cognitive load captured finer cognitive ability in APP^NL-G-F^ mice, which was otherwise masked in the standard task (Mehla et al., 2019a). Here, we report a paradigm that can sensitively capture episodic-like memory deficit in young APP^NL-G-F^ mice before significant Aβ plaque deposition.

Episodic memory in humans is a type of long-term memory that captures details of past experiences. However, assessing episodic memory in non-humans has been challenging due to the focus on autonoetic consciousness, the ability to travel back in time mentally to relieve past experiences (Tulving, 2005). Hence, a simple ‘what–where–when’ criterion was proposed to define episodic-like memory in non-humans (Clayton and Dickinson, 1998). Particularly, the ‘when’ component probes into the species’ capacity to remember back in time and mentally retrace trajectory through previously visited locations (‘where’) and the associated contents (‘what’). So far, no studies have evaluated episodic-like memory in APP^NL-G-F^ mice. Our study uses the ‘what–where–when’ framework to assess the ability of WT and APP^NL-G-F^ mice to recollect two distinct episodic-like representations. Unlike the use of inanimate cues, such as an object, location or context, to induce information binding and memory encoding to study episodic-like memory in AD mouse models (Savonenko et al., 2005; Davis et al., 2013a; Davis et al., 2013b), our paradigm uses an ethologically relevant social cue in competition with a non-social cue to induce learning and memory. We believe that the inclusion of a social cue in memory-testing paradigms can encourage spontaneous learning and more robust memory retrieval without the need for prolonged exposures to the cues or explicit training (Fellini and Morellini, 2013). In addition, unlike the other ‘what–where–when’ episodic-like memory tasks conducted in mice in which memory retrieval was tested after a short learning-to-recall interval (Dere et al., 2005a,b), our paradigm evaluated long-term memory retention after a 24 h interval. Hence, our paradigm encompasses incidental learning and evaluation of long-term memory. These features closely mimic characteristics of human episodic memory. Furthermore, our paradigm can delineate whether a specific component, or the complete episodic information, is impaired in mice. Specifically, the exploratory behavior during the recall tests represents the expression of spatial (‘what–where’) memory of past episodes. On the other hand, the scent-marking responses, which could only be induced by the memory for a female mouse but not the object, correspond to the expression of temporal (‘what–when’) memory. Expression of these two behaviors during the recall tests will represent intact retrieval of ‘what–where–when’ information or episodic-like memory.

Based on the behavioral responses of the mice during the recall tests, 3-month-old WT mice can form ‘what–where–when’ episodic associations of their past encounters and subsequently deploy these conjunctive representations during memory retrieval. In contrast, age-matched APP^NL-G-F^ mice showed a partial deficit in episodic-like memory retrieval, where only the recollection of ‘what–where’ target locations was perturbed. This study is the first to evaluate episodic-like memory in APP^NL-G-F^ mice and to identify a deficit as early as 3 months of age, when Aβ deposition was not aggressive. The memory deficit in 3-month-old APP^NL-G-F^ mice presages broader deficits in older age. Our study shows that episodic memory is a vulnerable cognitive function in aging, as 8-month-old WT mice displayed a deficit in recollecting the conjunctive ‘what–where–when’ representation of one episode associated with the female mouse. The finding is in agreement with episodic memory being one of the earliest memory systems to decline with physiological aging in humans (Tromp et al., 2015). With the 8-month-old WT mice already exhibiting episodic-like memory deficit, age-matched APP^NL-G-F^ mice displayed a similar, but otherwise slightly more aggressive, phenotype: these mice displayed compromised ability to recall two ‘what–where–when’ episodic representations associated with the object and the female mouse. The observations from the 8-month-old compared to the 3-month-old APP^NL-G-F^ mice highlights that the spatial component of episodic memory is more vulnerable to decline than the temporal component during AD progression.

The prefrontal cortex and hippocampus are closely linked anatomically, forming a neural circuit for learning and memory (Preston and Eichenbaum, 2013). Particularly, both regions are important for encoding and retrieval of episodic memory (Eichenbaum, 2017). Alterations to the neural activity in the prefrontal cortex and hippocampus have been reported in APP^NL-G-F^ mice. An increase in prefrontal–hippocampal network synchronicity was reported in 3-month-old APP^NL-G-F^ mice before prominent plaque deposition (Latif-Hernandez et al., 2019). Another study revealed synaptic impairments in the prefrontal cortex of 3- to 4-month-old APP^NL-G-F^ mice that were further aggravated and extended to the hippocampus of 6- to 8-month-old animals (Latif-Hernandez et al., 2020). In 8-month-old APP^NL-G-F^ mice, altered microstructure and glutamatergic-dependent gamma oscillations were detected in the mPFC (Pervolaraki et al., 2019). Our study similarly reported an alteration to specific prefrontal–hippocampal neuronal activity that was associated with poor episodic-like memory retrieval in APP^NL-G-F^ mice. c-Fos staining revealed neuronal hyperactivity in the mPFC and CA1 dorsal hippocampus in 3-month-old APP^NL-G-F^ mice compared to the age-matched WT mice. Interestingly, 8-month-old APP^NL-G-F^ mice only exhibited neuronal hyperactivity in the CA1 dorsal hippocampus compared to the age-matched WT mice. We postulate that the apparent lack of neuronal hyperactivity in the mPFC of 8-month-old APP^NL-G-F^ mice was due to the already higher c-Fos expression in the age-matched WT animals, which was also preserved in the 8-month-old APP^NL-G-F^ mice.

It is interesting how, in the absence of significant Aβ amyloidosis and other neuropathology (Masuda et al., 2016; Whyte et al., 2018; Latif-Hernandez et al., 2019; Mehla et al., 2019b; Saito et al., 2014), neural changes and memory decline are apparent in 3-month-old APP^NL-G-F^ mice. It also remains obscure how neuronal hyperactivation in specific prefrontal–hippocampal regions relates to memory alteration in these young animals. However, strong evidence suggests that soluble forms of Aβ are responsible for neuronal hyperactivity and dysfunction in the early stages of AD without plaque pathology, thereby contributing to memory impairment in young animals (Hector and Brouillette, 2021). Moreover, findings from AD mouse models and human patients highlight that neuronal circuits are hyperactive in the early stages of AD (Busche and Konnerth, 2015). For example, in the APP23/PS45 transgenic AD model, massive neuronal hyperactivity was observed in the CA1 hippocampus without amyloid plaque deposition (Busche et al., 2012). Similarly, elevated blood-oxygen-level-dependent (BOLD) functional magnetic resonance imaging revealed abnormal hippocampal hyperactivity in preclinical AD patients (McDonough et al., 2020; Dickerson et al., 2005). The aberrant neuronal hyperactivity can induce widespread neural network dysfunction and drive cognitive impairments before the onset of neurodegeneration (Busche and Konnerth, 2015). We proposed that a similar sequence of events is happening in the 3-month-old APP^NL-G-F^ mice: in the absence of plaque pathology, the soluble Aβ peptides underlie abnormal neuronal excitability in the mPFC and CA1 dorsal hippocampus to drive the deficit in recollecting spatial information of past episodes. Subsequently, the memory impairment becomes more aggravated in 8-month-old APP^NL-G-F^ mice as amyloid plaque pathology develops (Saito et al., 2014; Masuda et al., 2016; Whyte et al., 2018; Latif-Hernandez et al., 2019; Mehla et al., 2019b).

### Limitations

Although our study sensitively detects episodic-like memory deficits and reported neuronal activities that go awry in APP^NL-G-F^ mice, there are a few limitations and unaddressed questions to our work. First, given the availability of only homozygous APP^NL-G-F^ mice in our laboratory, we were unable to generate the corresponding control littermates. Instead, we used C57BL/6J mice with birthdates that matched the APP^NL-G-F^ as control mice. However, the choice to use C57BL/6J mice as a control for the APP^NL-G-F^ mice is not peculiar as the same control was also chosen in other studies (see summary in Sakakibara et al., 2019). Next, our paradigm does not allow proper separation between episodic-like memory acquisition, consolidation and retrieval. Although the episodic-like memory deficits observed in the APP^NL-G-F^ and 8-month-old WT mice were reported as impaired memory retrieval, there could be a problem in memory encoding that our paradigm cannot decipher. Third, we only assessed for neuronal activity in the mPFC and hippocampus after the episodic-like memory retrieval task. However, neuronal hyperactivity could already be present in resting-state APP^NL-G-F^ mice. An extra experiment to compare the c-Fos staining between resting-state animals and animals that have gone through the behavioral task is apt to substantiate whether the neuronal activation is indeed induced by the episodic-like memory task. Fourth, our c-Fos analysis did not allow us to compare 3- and 8-month-old WT animals to statistically conclude how neuronal activities were perturbed with age. However, we did observe subtle neuronal hyperactivity in the mPFC regions of 8-month-old WT mice compared to the 3-month-old animals. This change in neural activity could potentially account for the episodic-like memory deficit seen in the older WT mice. Lastly, we would like to highlight that impairment in human episodic memory is not limited to the inability to recall a few episodes but multiple episodic memories. Our paradigm only evaluated the memory of two episodes in mice. More complex tasks that require the mice to remember multiple items in context (Panoz-Brown et al., 2016; Roberts, 2016) or order of events (Panoz-Brown et al., 2018; Wright, 2018; Zentall, 2019) would more faithfully recapitulate features of human episodic memory.

## Conclusions

The APP^NL-G-F^ mouse model is a preclinical model to study incipient pathologies during the early stages of AD to facilitate effective interventions. However, detecting cognitive impairments in the early disease phase has been challenging. Our study is the first to investigate episodic-like memory in the APP^NL-G-F^ mouse model. It reveals that APP^NL-G-F^ knock-in accelerates age-dependent episodic-like memory decline such that the impairment is discernible in early disease development. Our work also shows that poor episodic-like memory retrieval in APP^NL-G-F^ mice is associated with specific mPFC and hippocampus neuronal hyperactivity. These findings parallel the observations in patients, where episodic memory decline is one of the earliest hallmarks of AD, and hyperactivity in the prefrontal cortex and hippocampus was detected during the prodromal phase in patients with mild cognitive impairment (McDonough et al., 2020). Thus, a longitudinal decline in episodic memory in cognitively normal adults, or clinical imaging for aberrant neuronal excitability in the prefrontal–hippocampal network, could be a non-invasive readout of AD. The ability to predict future progression into AD during the prodromal phase will provide an early therapeutic window to direct interventions before the onset of neurodegeneration.

## MATERIALS AND METHODS

### Animals

Homozygous APP^NL-G-F^ mice were obtained from a breeding colony at the Nanyang Technological University vivarium. Age-matched WT C57BL/6J were procured from a local supplier (InVivos, Singapore) and designated as the control mice. Behavior tests were conducted in 3- and 8-month-old male mice. None of the male mice had contact with a female conspecific after weaning. A 3-month-old WT C57BL/6J female mouse served as the social stimulus during the experience phase. Animals were maintained in a vivarium with an inverted 12:12 h light-dark cycle (light on at 21:00; light off at 09:00), with *ad libitum* access to food and water. All experimental procedures were reviewed and approved by the Institutional Animal Care and Use Committee (IACUC) of Nanyang Technological University (IACUC #A19012).

### Apparatus

Behavioral tests were performed in a dark room illuminated by red light. The apparatus consisted of a Plexiglas 40×40×40 cm square box with red walls. The base was layered with 135gsm white paper to capture the scent marks. One of the four walls of the arena was lined with geometric cues (striped wall) to allow spatial orientation in the arena (Fellini and Morellini, 2011). The arena was subdivided into two equal compartments by a black polycarbonate wall. A 5×5 cm square hole was created at the base of the wall to allow the mice to cross between the two compartments. Two 6×6×15 cm chambers made of metal mesh were positioned inside the arena, with one in the corner of the left compartment and the other in the corner of the right compartment (Fig. S1A).

### Episodic-like memory behavioral paradigm

The paradigm was modified from a previous study (Fellini and Morellini, 2013). Episodic-like memory was defined as the mice's ability to form and recall conjunctive ‘what–when–where’ episodic associations based on their past encounters with an object or a female mouse (‘what’), at a specific location in the arena (‘where’), and at a specific time (‘when’) (Fig. 1A). In brief, mice with intact episodic-like memory retrieval would spend more time around the target-associated zone compared to the opposing empty zone in the arena during the recall tests (‘what–where’ memory). In addition, the mice would scent mark more in the diurnal phase associated with the female mouse (‘what–when’ memory) during the recall tests. Expression of these two behaviors will constitute an intact ‘what–where–when’ episodic-like memory retrieval.

#### Experience phase

The mice went through 2 days of experience phase. Each phase consisted of a morning and a night trial. Before the trials, home cages were placed in the behavior suite for 1 h to habituate the mice to the room. After the habituation period, the mice underwent a 10 min trial in the morning (between 11:00 and 12:00) and a 10 min trial at night (between 19:00 and 20:00). In the morning, an inanimate object was placed inside one of the chambers in the arena and the other chamber was left empty. At night, a female mouse was placed inside one of the chambers (in the same position as the earlier object) and the opposing chamber was left empty. At the start of each trial, experimental male mice were positioned in the arena with the nostrils contacting the object or female mouse chamber. Subsequently, the mice were allowed to explore the arena freely. At the end of the 10 min, the mice returned to their home cages. The mice were run in a consistent order for the same arena to maintain olfactory cues for the following mouse. After all the mice had gone through the trials for the morning or night session, the home cages were returned to the vivarium. The arenas were then thoroughly cleaned using 70% ethanol. To prevent odor crossover from the female mouse, two identical sets of chambers were prepared, one for the object and the other for the female mouse. Counterbalancing was done for (1) the time of the day the object and female mouse appeared, and (2) the location of the object and female mouse in the arena.

#### Recall tests

On the third day, the mice underwent a 10 min recall test in the morning and at night. The behavioral setup and procedure were the same as the experience trials, except that the object or the female mouse was absent in the chamber (i.e. both chambers in the arena were empty). Another identical set of arenas and chambers was used to prevent the presence of female mouse odor in the recall tests. At the end of the night recall test, experimental mice were returned to their respective home cages and were left undisturbed.

#### Subject groups

A control group comprised of 3- to 5-month-old WT C57BL/6J mice (*n*=7) underwent the behavior routine without going through the experience trials and recall tests. These control mice were habituated to the behavior suite but remained in their home cages during the experience trials and recall tests. Four experimental groups comprised of 3-month-old WT (*n*=27), 3-month-old APP^NL-G-F^ (*n*=29), 8-month-old WT (*n*=19) and 8-month-old APP^NL-G-F^ (*n*=13) mice underwent the complete behavior routine. Sample sizes were defined based on the availability of mice.

### Behavioral analysis

Cameras were mounted 80 cm above the arena. ANY-maze™ Video Tracking System software (Stoelting Co.) was used to record the exploratory behaviors of mice. The middle point of the mouse body was used as a reference point. Exploratory behavior was quantified by the time spent by the mice around the chambers (6 cm away from each side of the chamber exposed to the mice) (Fig. S1B). This area was designated as the zone. Contrast ratios corresponding to the proportion of time spent around the target zone relative to the opposing empty zone were calculated for the experience trials and recall tests [i.e. (time_object_/time_empty_) and (time_female mouse_/time_empty_)]. Scent-marked papers were sprayed with ninhydrin (Sigma-Aldrich, N1286) and allowed to dry for 30[Supplementary-material sup1]min. Scent marking was quantified using a method previously described (Arakawa et al., 2008a). Scent marks were scored in the compartment of the arena associated with the object or the female mouse in the experience trials and recall tests (Fig. S1C). All animals were included in the time spent and scent-marking analyses.

### Immunohistology

Animals were anesthetized with isoflurane 90[Supplementary-material sup1]min after the night recall test. Transcardial perfusion was first carried out with 75 ml phosphate-buffered saline (PBS) followed by 50 ml of 4% paraformaldehyde (PFA; dissolved in PBS). Harvested brains were kept overnight in 4% PFA at 4°C before being transferred to 30% (w/v) sucrose for 48 h. Serial coronal sections of 40 μm thickness were cut on a cryostat (Leica, CM1950) from the olfactory bulb to the posterior hippocampus. Free-floating sections were stored at −20°C in an anti-freeze solution (buffered saline:ethylene glycol:glycerol=2:1:1).

Brain structures were anatomically defined according to the Paxinos and Watson atlas (Paxinos and Franklin, 2001). Brain sections around bregma −1.70 mm were stained with anti-Aβ_1-42_ antibody (Abcam, ab201060; 1:1000) to assess the Aβ pathology. Brain sections around bregma 1.98 mm (mPFC), −1.70 mm (dorsal hippocampus) and −3.28 mm (ventral hippocampus) were stained with anti-c-Fos antibody (Invitrogen, A48282; 1:10,000) to assess recent neuronal activity. The staining protocol was as follows: brain tissue sections were washed in 1× PBS (4×5 min) and incubated in blocking buffer [5% w/v bovine serum albumin (BSA) with 0.5% v/v Triton X-100 in PBS] for 1 h at room temperature. The sections were then incubated with diluted primary antibody [in 5% w/v BSA with 0.5% v/v Triton X-100 in Tris-buffered saline (TBS)] for 24 h at room temperature. Tissue sections were subsequently washed in 1× PBS (4×5 min) and incubated with Alexa Fluor Plus 594 goat anti-rabbit secondary antibody (Invitrogen, A11012; 1:1000) (in 5% w/v BSA with 0.5% v/v Triton X-100 in TBS) for 2 h at room temperature in the dark. Tissue sections were then washed in 1× PBS (4×5 min) and mounted onto Superfrost microscope slides (Fisher Scientific, 22-037-246) with ProLong Gold Antifade Mountant with DAPI (Invitrogen, P36931).

### Microscopy and analysis

Images were acquired with a Zeiss Z1 Axio Observer inverted fluorescence microscope using a 10× objective lens with 1.6× digital magnification (total optical magnification=16×). Identical exposure time was used to acquire images across the different brain samples. The experimenter was unaware of the animal groups when acquiring the images. ImageJ was used to quantify the Aβ levels and the number of c-Fos-positive nuclei. Briefly, an intensity threshold was used to highlight the Aβ and the c-Fos signals. The threshold was kept consistent across all images analyzed. The area of Aβ was quantified in the cortex and dorsal hippocampus (CA1, CA3, DG). The number of c-Fos-positive nuclei was quantified in the mPFC (ACC, PL-IL), dorsal hippocampus (CA1, CA3, DG) and ventral hippocampus (CA1, CA3). Counting frames were based on the entire imaged region (for Aβ) and the shape of the brain structures (for c-Fos). Aβ quantification was based on the percentage of Aβ area per region of interest. c-Fos quantification was based on the number of c-Fos*-*positive nuclei per region of interest. *n*=8 in each animal group were selected for Aβ staining and analysis. All animals were included in the c-Fos staining, but only 3-month-old WT (*n*=22), 3-month-old APP^NL-G-F^ (*n*=24 for ACC, *n*=25 for remaining brain regions), 8-month-old WT (*n*=19) and 8-month-old APP^NL-G-F^ (*n*=12) were included in the analysis. Some animals were excluded owing to technical failure when processing the samples.

### Statistical analyses

Each individual animal was used as a biological replicate. Statistical significance for the time spent near the target zone compared to the opposing empty zone in the experience trials and recall tests were analyzed with R (https://www.r-project.org/) using GLMM with gamma distribution. Statistical significance for the scent marks in the compartment associated with the object compared to the female mouse was analyzed using Wilcoxon signed rank test with Prism (GraphPad). Intergroup differences for Aβ were analyzed using one-way ANOVA with Tukey's HSD post hoc with Prism. Differences in c-Fos between WT and age-matched APP^NL-G-F^ mice were analyzed using non-parametric Mann–Whitney *U-*test with Prism. *P*≤0.05 was considered significant. Data used for analysis can be found in Dataset 1.

## Supplementary Material

10.1242/dmm.049945_sup1 Supplementary information
