# Supplementary material for: Impaired episodic-like memory in a mouse model of Alzheimer's disease is associated with hyperactivity in prefrontal–hippocampal regions
Source: Dis Model Mech. 2023 Mar 10;16(3):dmm049945. doi: 10.1242/dmm.049945 (PMC10040242; doi:10.1242/dmm.049945)
Supplement: Supplementary information [file dmm-16-049945-s1.pdf]

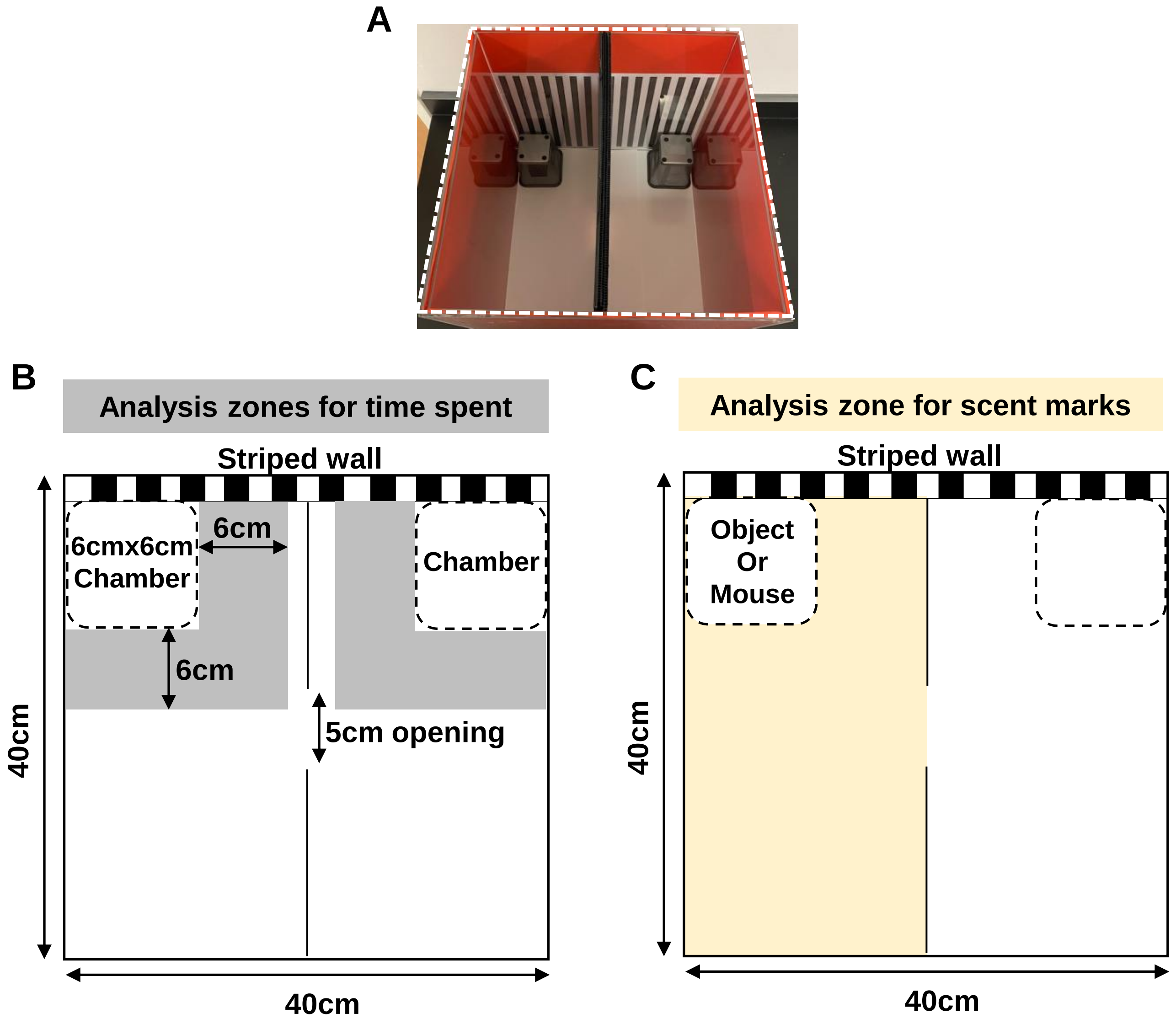

Fig. S1. Apparatus and analysis zones for the exploratory and scent marking responses.(A) Picture of the apparatus setup in the arena. (B) Exploratory behavior was determined by the time spent by the mice in the grey zones, defined as 6cm away from the chamber, in both the left and right compartments. (C) Scent marks were tabulated in the yellow region, in the compartment containing either the object or female mouse. Apparatus was not drawn to scale.

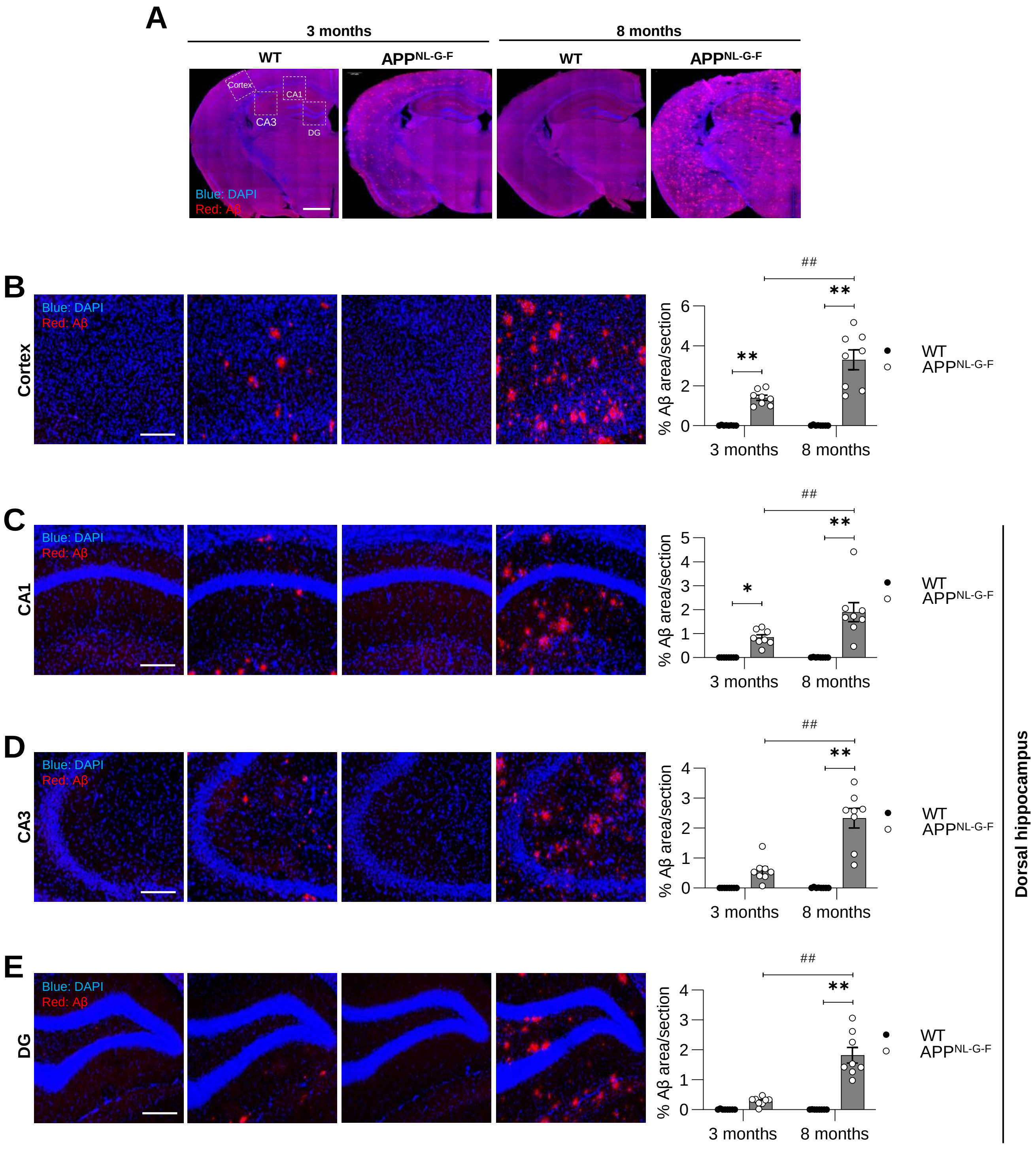

Fig. S2. Age-dependent Aβ deposition in the brain of APP$^{NL\text{-}G\text{-}F}$ mice. (A) Mouse brain coronal sections (bregma -1.70mm) with Aβ (red) and DAPI (blue) staining. White dotted boxes indicate the regions of interest. Scale bar = 1mm. (B-E) Percentage of Aβ area per region of interest for the (B) cortex, and (C) CA1, (D) CA3, and (E) DG of dorsal hippocampus. Left: Representative images of Aβ (red) and DAPI (blue) staining for all regions of interest. Scale bar = 200µm. Right: Quantification of the percentage area occupied by Aβ for each region of interest. All values are mean ± S.E.M. Differences are significant for $^{*}p \leq 0.05$ and $^{**/\#\#}p \leq 0.01$ (one-way ANOVA followed by Tukey's HSD *post-hoc*). 3-month-old WT (n = 8), 3-month-old APP$^{NL\text{-}G\text{-}F}$ (n = 8), 8-month-old WT (n = 8), 8-month-old APP$^{NL\text{-}G\text{-}F}$ (n = 8).

Disease Models & Mechanisms • Supplementary information

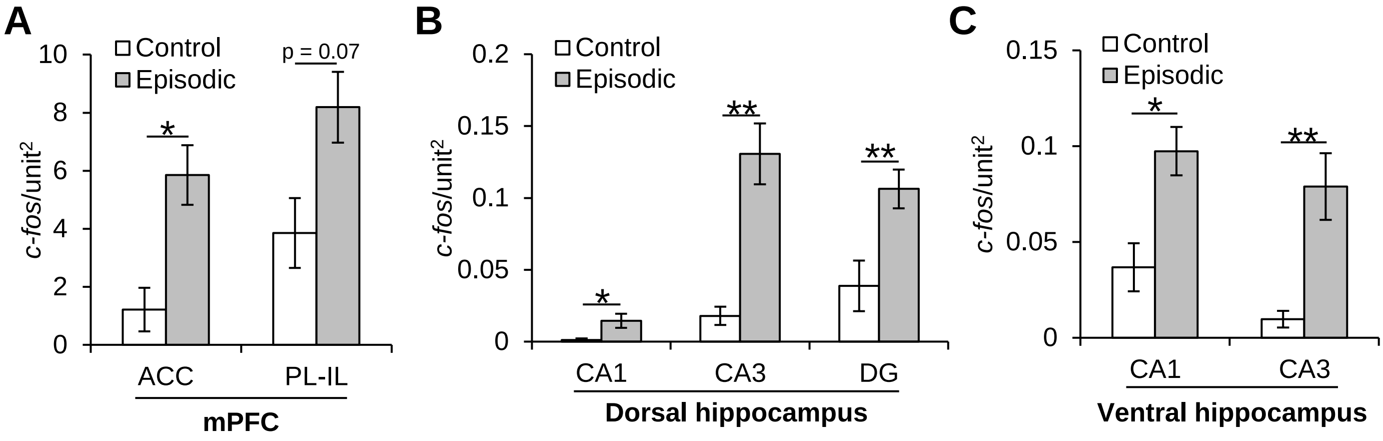

Fig. S3. Activation of mPFC and hippocampus during episodic-like memory retrieval in C57BL/6J mice. (A-C) Number of *c-fos* puncta per unit area in the (A) ACC and PL-IL of mPFC, (B) CA1, CA3, and DG of dorsal hippocampus, and (C) CA1 and CA3 of ventral hippocampus. 'Control' refers to mice that went through the behavior routine minus the experience trials and recall tests (see 'Materials and Methods' section); 'Episodic' refers to mice that went through the compete behavior routine. The control group comprised of 3-5 months old C57BL/6J WT mice (n = 7). The episodic group is the 3-month-old WT mice and the data is also used in Fig. 5 (n = 22). All values are mean ± S.E.M. Statistical analysis was performed with Mann-Whitney U test and significant against control for *p ≤ 0.05 and **p ≤ 0.01.

Dataset 1. Datasets used for analysis.

Click here to download Dataset 1
